# Improving Electronic Survey Response Rates Among Cancer Center Patients During the COVID-19 Pandemic: Mixed Methods Pilot Study

**DOI:** 10.2196/30265

**Published:** 2021-08-06

**Authors:** Cassandra A Hathaway, Melody N Chavez, Mika Kadono, Dana Ketcher, Dana E Rollison, Erin M Siegel, Anita R Peoples, Cornelia M Ulrich, Frank J Penedo, Shelley S Tworoger, Brian D Gonzalez

**Affiliations:** 1 Department of Cancer Epidemiology Moffitt Cancer Center Tampa, FL United States; 2 Participant Research, Interventions, and Measurements Core Moffitt Cancer Center Tampa, FL United States; 3 The AltaMed Institute for Health Equity Los Angeles, CA United States; 4 Department of Health Outcomes and Behavior Moffitt Cancer Center Tampa, FL United States; 5 Huntsman Cancer Institute Salt Lake City, UT United States; 6 Department of Population Health Sciences University of Utah Salt Lake City, UT United States; 7 Departments of Medicine and Psychology University of Miami Coral Gables, FL United States; 8 Department of Epidemiology Harvard T.H. Chan School of Public Health Boston, MA United States

**Keywords:** response rates, electronic survey, cancer, COVID-19, pandemic, surveillance, cancer patients, health promotion, digital health, patient experience, health outcomes

## Abstract

**Background:**

Surveys play a vital role in cancer research. During the COVID-19 pandemic, the use of electronic surveys is crucial to improve understanding of the patient experience. However, response rates to electronic surveys are often lower compared with those of paper surveys.

**Objective:**

The aim of this study was to determine the best approach to improve response rates for an electronic survey administered to patients at a cancer center during the COVID-19 pandemic.

**Methods:**

We contacted 2750 patients seen at Moffitt Cancer Center in the prior 5 years via email to complete a survey regarding their experience during the COVID-19 pandemic, with patients randomly assigned to a series of variations of prenotifications (ie, postcard, letter) or incentives (ie, small gift, modest gift card). In total, eight combinations were evaluated. Qualitative interviews were conducted to understand the level of patient understanding and burden with the survey, and quantitative analysis was used to evaluate the response rates between conditions.

**Results:**

A total of 262 (9.5%) patients completed the survey and 9 participated in a qualitative interview. Interviews revealed minimal barriers in understanding or burden, which resulted in minor survey design changes. Compared to sending an email only, sending a postcard or letter prior to the email improved response rates from 3.7% to 9.8%. Similarly, inclusion of an incentive significantly increased the response rate from 5.4% to 16.7%, especially among racial (3.0% to 12.2%) and ethnic (6.4% to 21.0%) minorities, as well as among patients with low socioeconomic status (3.1% to 14.9%).

**Conclusions:**

Strategies to promote effective response rates include prenotification postcards or letters as well as monetary incentives. This work can inform future survey development to increase response rates for electronic surveys, particularly among hard-to-reach populations.

## Introduction

Surveys are a critical aspect of many research studies, and electronic surveys are increasingly being used in research. Benefits of electronic compared to paper surveys include greater reach, higher survey completeness, lower costs, flexibility in survey design, real-time data access, and increased willingness of participants to share information [1-4]. Prior work has demonstrated that the vast majority of research participants, including cancer patients, prefer a computer-assisted survey compared to a paper-based survey [2,5,6].

Nevertheless, compared with mailed or in-person paper surveys, electronic surveys tend to have lower response rates and decreasing response rates over time [7-10], although most studies using paper surveys also experience attrition with follow-up [11-15]. Response rates, regardless of survey type, are usually lower in minority racial and ethnic groups, as well as among those with poorer health status, lower incomes, and lower education [16]. A study among breast cancer patients found that those who were older, had lower education levels, and had worse quality of life were more likely to prefer paper-based surveys to collect health data, indicating a potential barrier to electronic surveys in these populations [17].

As a consequence of the COVID-19 pandemic, there is reduced face-to-face interaction with research participants, increasing the need to reach study participants using remote approaches [18,19]. Thus, it is critical to evaluate potential approaches to engage participants and enhance the response to electronic surveys. For example, shortening the length of the survey and improving the clarity of questions can reduce the burden and improve understanding, leading to higher response rates [10,20-23]. Additionally, monetary and nonmonetary incentives, a notification prior to administering the survey, including an image in the email, and follow-up contact may also enhance participation [4,10,21,22]. To address the growing need to enhance response rates for electronic surveys, we used a mixed methods approach to (1) assess participant burden and understanding through qualitative interviews, and (2) quantitatively evaluate the impact of prenotifications and incentives on response rates of an electronic survey during the COVID-19 pandemic among individuals who were seen at a cancer center.

## Methods

### Study Population

This study included patients at Moffitt Cancer Center who were seen between January 1, 2015 and September 13, 2020; had English as a preferred language; were between 40 and 89 years old; lived in the cancer center catchment area; had a valid email address; and a last known vital status of alive. Half of the patients in this study had previously consented to an institutional biobanking study (Total Cancer Care: MCC14690, Advarra IRB Pro00014441; Moffitt Cancer Center Screening and Prevention Study: MCC14453, USF IRB 103792). We randomly selected patients for each pilot condition with oversampling of Hispanic and Black/African American patients. Similar to the general Moffitt Cancer Center population, the participants included in this study were those diagnosed with invasive and in situ cancer, benign diseases, and patients who were screened without a cancer diagnosis. The survey contained questions regarding COVID-19–specific behaviors, testing, symptoms and treatment, demographics, medical history, health behaviors, and psychosocial well-being (143 total items across 26 web pages). Participants were able to change their answers through a “back” button if desired. The survey was tested with staff members before sending to participants to check for usability, technical functionality, and appropriate wording. After surveys were submitted, they were reviewed by study staff for completeness.

### Ethical Statement

This study was approved by Advarra Inc (MCC 20629, Pro00043372). Emails invited eligible patients to the study and included a unique link to an information and consent page. This page included a description of the study goals, the approximate length of the survey, a Health Insurance Portability and Accountability Act authorization if they were not consented to a biobanking study or information about the biobanking study they had consented to previously, and the Institutional Review Board contact information. At the bottom of this web page was a unique link, based on the patient’s email address, to start the voluntary survey.

### Pilot Conditions

We evaluated eight different conditions with an email sent with a survey link for each condition, and various methods of prenotifications and incentives were tested based on findings from prior literature [10,20-23]. Although prior studies have shown that prenotifications and incentives improve response rates, there has been little work performed in this regard with cancer center patients, especially during the pandemic; therefore, we considered multiple methods and their combinations. Participants in the first condition (n=1000) received a lengthy (380-528 words) text-only email discussing the aim of the study, study procedures, and links for more information about the COVID-19 pandemic and the cancer center’s response. Due to cost and time constraints, the subsequent conditions included 250 patients each. Participants in conditions 2-8 received a condensed version of the email sent in condition 1, containing only a few sentences (119-142 words), with the cancer center’s logo and an image of the principal investigators’ signatures. Participants in condition 2 received only the condensed email, those in condition 3 received a mailed letter from the principal investigators of the study and the center’s Associate Center Director of Clinical Science, which discussed the importance of the work and noted that an email with the survey link would be sent shortly; this was followed by a condensed email 3 to 4 days after mailing the letter. Condition 4 was the same as condition 3 but with the addition of a small gift (Moffitt-branded adhesive phone wallet) in the envelope. Participants in condition 5 received a postcard about the study and asked patients to look for an email with a survey link, which was sent 3 to 4 days later. Participants in condition 6 received only the condensed email with an additional note stating that an electronic US $10 gift card would be sent via email within 5 days of completion of the survey. Participants in condition 7 received the same letter as sent in condition 3, further noting a US $10 gift card incentive upon survey completion. Participants in the final condition received a postcard noting that an email will be sent with a US $10 gift card after completing the survey. For all conditions, up to two reminder emails were sent in 4-day intervals.

### Covariates

We collected information on current age (continuous), years since their most recent visit to the cancer center (<2 years, 2-5 years), gender (male, female), race (White, non-White), ethnicity (non-Hispanic, Hispanic), cancer status (invasive cancer, benign, in situ, or no cancer diagnosis), and zip code to assess the area deprivation index (ADI) decile rank for the state of Florida, which ranges from 1 to 10. The ADI ranks neighborhoods based on socioeconomic factors, including income, education, employment, and housing quality, with a higher ADI rank indicating a greater socioeconomic disadvantage [24,25]. All variables were obtained through medical records and Cancer Registry data; missing information was supplemented with self-reported data from the survey where possible (eg, self-reported race and ethnicity). Data collected on the survey were linked to medical record data. All data were stored on a secured, password-protected server.

### Qualitative Interviews

Individual qualitative interviews were conducted with survey participants to better understand their motivations to participate in the survey, and to assess understanding of the survey questions and participant burden. Upon completion of the survey, participants within condition 1 (long email only) were asked if they would like to volunteer for an interview to provide feedback about their survey experience. A research coordinator contacted participants who volunteered and obtained verbal consent via telephone. Videoconference interviews (n=9) were scheduled an average of 4 weeks after participants completed the survey and were conducted by two trained interviewers (MC and MK). A semistructured interview guide was used with two primary domains: understanding and burden, informed by health literacy models and the perceived research burden literature [26-28]. The interviews were conducted over a period of 2 weeks using Zoom [29,30]. The interviews lasted an average of 21 minutes and were audio-recorded with participant consent. Data saturation was reached after nine interviews with participants in the first condition; therefore, we did not conduct interviews with the other pilot conditions.

### Qualitative Data Analysis

Interview transcripts were analyzed using rapid ethnographic methods [31,32] and constant comparison analysis [33], an integrative process of cumulative and concurrent data generation and analysis, to identify emergent themes that informed continuing data collection [34]. These methods were adopted to accommodate the time-sensitive nature of the research, since the survey was ongoing during analysis. Emergent themes were identified and agreed upon by the researchers, and when available, specific quotes that were representative of each theme were selected and segmented. Data saturation was reached after nine interviews (ie, no new themes emerged), consistent with other qualitative studies [35,36].

### Statistical Analysis

We calculated response rates for each pilot condition and compared groups of conditions (eg, pre-email notification vs none, incentive vs none) by calculating overall response rates as well as response rates within key sociodemographic groups. We used χ^2^ tests to assess statistical differences in response rates and logistic regression was used to estimate the odds of completing the survey between groups of conditions. We also used logistic regression analysis to assess the odds of response for each condition (compared to condition 2 with only the condensed email) adjusting for sociodemographic factors that were found to be significantly associated with response rates in univariable logistic regression. All *P* values were two-sided and analytic results were considered statistically significant if *P*<.05. Study data were collected and managed using REDCap electronic data capture tools hosted at Moffitt Cancer Center [37,38]. Analyses were performed using SAS version 9.4 (SAS Institute Inc).

## Results

### Population Characteristics

Among the 2750 patients contacted, a total of 262 patients (9.5%) completed the survey. Compared to the total invited population, those who completed the survey were slightly older, more likely to be female, less likely to be Black, and more likely to be Hispanic (Table 1). Those with higher measures of socioeconomic status (ie, a lower ADI rank) were also more likely to complete the survey (mean decile rank of 4.5 vs 5.0 among the invited population). Most patients had a cancer diagnosis (75%), approximately 7% of those contacted had a benign or in situ diagnosis, and 18% had no reported cancer diagnosis. The survey took an average of 18.4 minutes to complete. Demographic information for each pilot condition is shown in Multimedia Appendix 1.

**Table 1:** Demographic characteristics of participants who completed the survey and those who were invited to participate.

Characteristics		Completed survey (n=262)	Invited to survey (n=2750)
Time to complete survey (minutes), mean (SD)		18.4 (14.3)	N/A^a^
Age (years), mean (SD)		65.6 (10.9)	64.5 (11.6)
Years since last Moffitt visit, mean (SD)		1.0 (1.3)	1.5 (1.6)
Area Deprivation Index State Decile Rank, mean (SD)^b^		4.5 (2.6)	5.0 (2.7)
**Gender, n (%)**			
	Male	118 (45.0)	1339 (48.7)
	Female	144 (55.0)	1411 (51.3)
**Race, n (%)**			
	American Indian	0 (0)	4 (0.2)
	Asian/Pacific Islander	1 (0.4)	41 (1.5)
	Black	26 (9.9)	379 (13.8)
	Other	4 (1.5)	69 (2.5)
	White	231 (88.2)	2192 (79.7)
	Unknown	0 (0)	65 (2.4)
**Ethnicity, n (%)**			
	Hispanic	38 (14.5)	378 (13.8)
	Non-Hispanic	224 (85.5)	2312 (84.1)
	Unknown	0 (0)	60 (2.2)
**Cancer status, n (%)**			
	Invasive	212 (80.9)	2058 (74.8)
	Benign or in situ	9 (3.4)	193 (7.0)
	No cancer	41 (15.7)	499 (18.2)
**Stage at first diagnosis^c^, n (%)**			
	0	9 (4.3)	77 (3.7)
	1	64 (30.2)	476 (23.1)
	2	28 (13.2)	280 (13.6)
	3	16 (7.6)	183 (8.9)
	4	17 (8.0)	157 (7.6)
	Unknown	78 (36.8)	885 (43.0)
**Recruitment method, n (%)**			
	Long email only	26 (9.9)	1000 (36.4)
	Condensed email only	20 (7.6)	250 (9.1)
	Condensed email + letter	28 (10.7)	250 (9.1)
	Condensed email + letter + gift^d^	39 (14.9)	250 (9.1)
	Condensed email + postcard	21 (8.0)	250 (9.1)
	Condensed email + gift card	36 (13.7)	250 (9.1)
	Condensed email + letter + gift card	46 (17.6)	250 (9.1)
	Condensed email + postcard + gift card	46 (17.6)	250 (9.1)

^a^N/A: not applicable.

^b^Missing: n=11 completed survey, n=100 invited to survey.

^b^Among those diagnosed with invasive or metastatic cancer.

^c^The gift included a Moffitt-branded adhesive phone wallet inside the envelope.

### Qualitative Interviews

The qualitative interview responses were summarized using a priori determined themes (ie, understanding, burden) and emergent themes (ie, access, question-specific feedback). All participants reported being able to understand and comprehend most survey questions; however, participants also reported that if they did not understand the question, they skipped it. If participants were unable to answer the question accurately with the answers provided, they answered the best they could. When available, participants clarified their answers in a free-text field at the end of the survey and suggested adding free-text fields to some questions to allow participants to clarify their responses, which were then added to the survey for subsequent conditions.

Participants did not report experiencing stress due to the survey; however, some participants commented that the survey was too long. One participant who was not undergoing treatment said, “If I wasn’t feeling well, I’ll tell you this [survey] is the last thing I’d do.” Other participants mentioned the extra effort required to answer questions about their cancer history, such as recalling specific dates, diagnosis (ie, first, recurrence), treatments, and medications. Other burden-related comments included high levels of stress participants were experiencing in their lives (ie, due to cancer, COVID-19 pandemic) and feeling isolated. Given this initial feedback, several questions were removed or reworded in the survey to reduce participant burden.

Participants did not report difficulty accessing the survey, although interviewed participants were among those who successfully completed the survey and agreed to provide feedback. They felt that the email was clear and the links were easy to find. However, participants did provide specific suggestions related to improving access, including the use of text messages or the patient portal to notify participants that a survey was emailed. Most participants did not have feedback or recommendations to improve access to the survey.

### Condition Response Rates

Table 2 presents response rates for the overall sample and sociodemographic subgroups. The pilot condition with the lowest response rate was the long email only and the highest responses were for the conditions with a prenotification (either letter or postcard) and receiving a US $10 gift card for completing the survey. Further, differences in response rates were observed for the pilot conditions based on sociodemographic factors. For example, those receiving an email, postcard, and gift card had the highest response rates among non-White individuals and those with lower socioeconomic status, and participants of Hispanic ethnicity responded more frequently when receiving an email, letter, and gift card. Women had a higher response when a letter and an incentive were included, showing similar results if the incentive was a gift or a gift card. Alternatively, men responded more frequently when there was a postcard and a gift card, although responses were only slightly lower for the letter-only or the letter+gift card conditions.

Multivariable logistic regression was used to evaluate the odds of survey completion by pilot condition and sociodemographic factors. Compared with receiving only the condensed email, adding a letter and gift, a gift card, a letter and gift card, or a postcard and gift card significantly increased the odds of survey response (Table 3). Further, having previously consented to a Moffitt biobanking study versus not was related to a higher odds of survey response. Having a worse socioeconomic disadvantage (ADI rank 6-10 vs 1-5) as well as the last visit to the cancer center being more than 2 years from the date of the email led to a decreased response. Results were similar in the univariable models (data not shown).

**Table 2:** Response rates for each condition overall and by sociodemographic factor.

Sociodemographic factor			Long email only (n=1000)		Email only (n=250)		Email+letter (n=250)		Email+letter+gift (n=250)		Email+postcard (n=250)		Email+gift card (n=250)		Email+letter+ gift card (n=250)		Email+postcard+gift card (n=250)
Minutes to complete survey, mean (SD)			20.4 (21.1)		14.6 (9.6)		15.6 (7.0)		18.3 (11.5)		21.7 (22.0)		22.0 (18.6)		16.6 (7.1)		18.4 (14.3)
Overall response rate			2.6%		8.0%		11.2%		15.6%		8.4%		14.4%		18.4%		18.4%
**Race**																	
	White	3.1%		9.0%		11.7%		18.1%		8.9%		15.9%		19.0%		18.7%	
	Non-White	0.0%		2.6%		9.1%		4.3%		6.4%		8.2%		16.0%		17.3%	
**Ethnicity**																	
	Non-Hispanic	2.8%		8.1%		11.3%		16.8%		7.9%		15.6%		16.9%		17.0%	
	Hispanic	1.5%		7.4%		10.3%		8.3%		11.8%		7.9%		27.0%		25.0%	
**Gender**																	
	Male	1.7%		8.8%		15.2%		11.8%		5.8%		12.2%		15.4%		18.1%	
	Female	3.3%		7.4%		7.2%		20.2%		10.8%		16.8%		21.9%		18.7%	
**Years since last visit**																	
	<2	3.3%		8.1%		14.9%		17.3%		9.9%		17.4%		21.1%		20.4%	
	2-5	1.0%		6.9%		3.4%		12.2%		5.1%		7.7%		12.0%		13.0%	
**Age (years)**																	
	<65	2.8%		4.1%		10.8%		11.2%		5.6%		14.2%		21.1%		19.2%	
	≥65	2.5%		11.7%		11.5%		18.9%		10.6%		14.6%		15.7%		17.7%	
**Cancer status**																	
	Invasive cancer	3.0%		7.6%		14.0%		16.8%		9.8%		14.4%		17.8%		20.5%	
	Benign, in situ, or no cancer	1.2%		9.1%		5.1%		12.1%		4.5%		14.5%		20.8%		10.9%	
**ADI^a^ decile**																	
	1-5 (less disadvantaged)	2.9%		9.9%		13.8%		17.2%		12.0%		14.9%		19.2%		21.2%	
	6-10 (more disadvantaged)	1.9%		5.1%		6.5%		14.9%		3.1%		13.0%		19.1%		13.1%	

^a^ADI: Area Deprivation Index.

**Table 3:** Odds of completing the survey for the different pilot conditions and various sociodemographic factors.

Variables in multivariate model	OR^a^ (95%CI)	*P* value
Email + letter (vs short email only)	1.51 (0.81-2.82)	.20
Email + letter + gift (vs short email only)	2.29 (1.27-4.13)	.01
Email + postcard (vs short email only)	1.02 (0.53-1.99)	.95
Email + gift card (vs short email only)	2.03 (1.11-3.71)	.02
Email + letter + gift card (vs short email only)	2.83 (1.59-5.06)	<.001
Email + postcard + gift card (vs short email only)	2.55 (1.42-4.58)	.002
Previous consent to biobanking study (vs not consented)	2.15 (1.59-2.93)	.006
Area Deprivation Index decile (6-10 vs 1-5)	0.65 (0.48-0.88)	.01
Cancer status (invasive vs benign/in situ/no cancer)	1.04 (0.71-1.53)	.84
Age (per 10 years)	1.13 (0.99-1.29)	.08
Non-White (vs White/missing)	0.68 (0.45-1.04)	.08
Hispanic (vs non-Hispanic/missing)	1.11 (0.74-1.68)	.60
Years since last visit to Moffitt (2-5 vs <2)	0.52 (0.36-0.73)	<.001
Female (vs male)	1.33 (0.98-1.81)	.06

^a^OR: odds ratio.

Logistic regression analyses were performed to examine the impact of including a prenotification and/or incentive among sociodemographic subgroups. Compared with receiving only an email, response rates were significantly better among those receiving a prenotification letter or postcard (Table 4). A significant increase in response was observed with the prenotification for nearly every sociodemographic group examined, except those last seen at the cancer center more than 2 years ago, those without invasive cancer, and those with a worse socioeconomic disadvantage. The largest increase in response rates was observed for Hispanic and non-White patients. Further, when comparing no incentive to any incentive (gift card or gift), the response rate increased from 5.4% to 16.7% overall; every group had significantly improved response rates (Table 5). The largest increases in response rates were for non-White individuals, those with a greater socioeconomic disadvantage (ADI=6-10), and those without an invasive cancer diagnosis (Table 5). The condensed email also had significantly higher response rates; generally, there were no differences in response rates when comparing the two different prenotification modalities (letter vs postcard) or incentive types (gift vs gift card) (see Multimedia Appendix 2). Overall, 192 patients (7% of 2750) read the consent and answered at least one question but did not complete the survey. The noncompletion rate of the survey was the highest in conditions 1 and 2 (10.9% and 10.8%, respectively) and was the lowest for conditions 6, 7, and 8 (0.8%, 1.2%, and 0.8%, respectively) (data not shown).

**Table 4:** Response rates and odds of response when including a pre-email notification letter or postcard overall and by sociodemographic groups.

Socioeconomic group	Long or condensed email only (n=46/1250)		Condensed email + letter or postcard (n=49/500)		OR^a^ (95% CI)
	Complete, n (%)	Incomplete, n (%)	Complete, n (%)	Incomplete, n (%)	
Overall	46 (3.7)	1204 (96.3)	49 (9.8)	451 (90.2)	2.84 (1.87-4.31)
White	45 (4.3)	1000 (95.7)	42 (10.3)	367 (89.7)	2.54 (1.64-3.94)
Non-White	1 (0.5)	204 (99.5)	7 (7.7)	84 (92.3)	17.00 (2.06-140.31)
Non-Hispanic	42 (3.9)	1048 (96.2)	42 (9.6)	395 (90.4)	2.65 (1.70-4.13)
Hispanic	4 (2.5)	156 (97.5)	7 (11.1)	56 (88.9)	4.88 (1.37-17.29)
Male	18 (3.1)	557 (96.9)	26 (10.6)	219 (89.4)	3.67 (1.97-6.84)
Female	28 (4.2)	647 (95.9)	23 (9.0)	232 (91.0)	2.29 (1.29-4.06)
<2 years since last visit	37 (4.3)	828 (95.7)	42 (12.5)	293 (87.5)	3.21 (2.02-5.09)
2-5 years since last visit	9 (2.3)	376 (97.7)	7 (4.2)	158 (95.8)	1.85 (0.68-5.06)
<65 years old	18 (3.0)	575 (97.0)	19 (8.3)	209 (91.7)	2.90 (1.50-5.64)
≥65 years old	28 (4.3)	629 (95.7)	30 (11.0)	242 (89.0)	2.78 (1.63-4.76)
Any cancer	37 (3.9)	902 (96.1)	42 (11.8)	313 (88.2)	3.27 (2.06-5.18)
Benign, in situ, or no cancer	9 (2.9)	302 (97.1)	7 (4.8)	138 (95.2)	1.70 (0.62-4.66)
ADI^b^ rank 1-5	30 (4.3)	664 (95.7)	38 (12.9)	256 (87.1)	3.29 (1.99-5.42)
ADI rank 6-10	13 (2.5)	503 (97.5)	9 (4.7)	181 (95.3)	1.92 (0.81-4.58)

^a^OR: odds ratio.

^b^ADI: Area Deprivation Index.

**Table 5:** Response rates and odds of response when including a pre-email notification of an incentive upon completion overall and by sociodemographic groups.

Socioeconomic group	No incentive (n=95/1750)		Any incentive (n=167/1000)			OR^a^ (95% CI)
	Complete, n (%)	Incomplete, n (%)	Complete, n (%)	Incomplete, n (%)		
Overall	95 (5.4)	1655 (94.6)	167 (16.7)	833 (83.3)	3.49 (2.68-4.55)	
White	87 (6.0)	1367 (94.0)	144 (17.9)	659 (82.1)	3.43 (2.59-4.55)	
Non-White	8 (2.7)	288 (97.3)	23 (11.7)	174 (88.3)	4.76 (2.08-10.87)	
Non-Hispanic	84 (5.5)	1443 (94.5)	140 (16.6)	705 (83.4)	3.41 (2.57-4.54)	
Hispanic	11 (4.9)	212 (95.1)	27 (17.4)	128 (82.6)	4.07 (1.95-8.47)	
Male	44 (5.4)	776 (94.6)	74 (14.3)	445 (85.7)	2.93 (1.98-4.34)	
Female	51 (5.5)	879 (94.5)	93 (19.3)	388 (80.7)	4.13 (2.88-5.93)	
<2 years since last visit	79 (6.6)	1121 (93.4)	133 (19.1)	563 (80.9)	3.35 (2.49-4.51)	
2-5 years since last visit	16 (2.9)	534 (97.1)	34 (11.2)	270 (88.8)	4.20 (2.28-7.75)	
<65 years old	37 (4.5)	784 (95.5)	78 (16.6)	392 (83.4)	4.22 (2.80-6.35)	
≥65 years old	58 (6.2)	871 (93.8)	89 (16.8)	441 (83.2)	3.03 (2.14-4.30)	
Any cancer	79 (6.1)	1215 (93.9)	133 (17.4)	631 (82.6)	3.24 (2.41-4.35)	
Benign, in situ, or no cancer	16 (3.5)	440 (96.5)	34 (14.4)	202 (85.6)	4.63 (2.50-8.58)	
ADI^b^ rank 1-5	68 (6.9)	920 (93.1)	104 (18.1)	470 (81.9)	2.99 (2.16-4.14)	
ADI rank 6-10	22 (3.1)	684 (96.9)	57 (14.9)	325 (85.1)	5.45 (3.28-9.07)	

^a^OR: odds ratio.

^b^ADI: Area Deprivation Index.

## Discussion

### Overview

In this study, patients seen at a cancer center who were sent a prenotification letter or postcard had higher response rates to an email invitation for an electronic survey than those not sent a prenotification, with much higher rates among those offered an incentive. Notably, both types of incentives—a small gift included with the prenotification letter or a gift card upon survey completion—improved response rates of electronic surveys for individuals who are often underrepresented in studies, including racial or ethnic minorities and those with low socioeconomic status. Further, prior engagement in biobanking studies and having been seen more recently at the cancer center were strong predictors of higher response rates. Finally, qualitative interviews identified that although the survey itself was not particularly burdensome, cancer patients are experiencing many external stressors due to the pandemic that may interfere with or deter from participation.

### Conclusions

Our study is consistent with previous literature showing that prenotifications can increase response rates, particularly for electronic surveys [4,10,20-22,39]. We also observed higher response rates when including a gift or monetary incentive; however, prior studies only observed an increase with monetary incentives [39-42] and not with other incentives [43-46]. Interestingly, in cancer patients, both a small gift for all invited individuals or a gift card for those who completed the survey led to similar response rates. Above and beyond the cost of sending the letters, postcards, and emails, we spent US $378 on gifts sent to all 250 invited patients in condition 4 and US $1280 on gift cards sent to the 128 participants who completed surveys in conditions 6-8. This yielded a cost of US $9.69 per completed survey for those in condition 4 and US $10 for those in conditions 6-8. Because these conditions had similar response rates and similar cost per completed survey, each study should evaluate the feasibility and best method for their population.

This study builds on the literature by finding higher response rates in traditionally underrepresented groups when sent a prenotification and/or incentive, which has not been evaluated previously. Additionally, patients with prior involvement in research and who had more recently been seen at the study site were more likely to have a higher response overall, indicating that connection or engagement with the study site in advance of the invitation to research studies could be a critical modality to enhance response rates for remote studies, especially during a pandemic such as COVID-19. The salient nature of this survey may have increased our response rates, although we are unable to evaluate this as participants in all pilot conditions received the same survey.

Ensuring understanding and minimizing burden are important in the development and dissemination of effective surveys for research. Although participants noted that the survey was lengthy, those who completed the survey expressed that they did not feel the survey was overly burdensome. Nevertheless, the length of the survey (approximately 15-20 minutes) may have been a barrier to participation for nonrespondents. Participant suggestions of how to further improve understanding and minimize burden included: (1) reducing the use of medical terminology and incorporating lay terms; (2) adjusting the questions to make the language more specific and less confusing (eg, define what “physical contact” means in the question “How often have you had physical contact with individuals that do not live with you?”); and (3) more clearly communicating the expectations and purpose of the survey through the consent process or via email (eg, length of the survey, expected time commitment). When possible, the length of the survey should be reduced to include questions focused on answering the primary research questions, which may increase response rates [47]. We used adaptive questions such that some questions were only shown if participants self-reported a cancer diagnosis, thereby reducing the overall burden.

### Strengths and Limitations

This study has many strengths, including the use of a mixed methods approach to improve the design of the survey and response rates. As a result of the qualitative interviews, adjustments were made to specific survey questions to improve understanding and the invitation email was substantially condensed to increase readability. We oversampled underrepresented groups to ensure adequate representation, allowing us to evaluate response rates within specific populations. However, due to low response rates with the first pilot condition (long email only), some analyses had limited power. Further, we only conducted qualitative interviews with those who completed the survey and answered the survey question asking whether they would be interested in participating in the interview, which limited our ability to understand why patients did not complete the survey. Our overall response rate was low (9.5%), which may be due to our population of older adults and the increased mortality rates among cancer patients, as well as barriers to accessing online surveys. However, 7% of those invited to participate started the study but did not finish. Future work should attempt to interview nonrespondents to understand the reason for nonparticipation and incomplete participation, which can help to determine strategies to address nonresponse.

### Implications

As the COVID-19 pandemic forces research to evolve, use of electronic surveys is increasing in lieu of in-person interactions [48]. The use of incentives and prenotifications can increase the response rates overall and in vulnerable populations, leading to more diverse studies, increased generalizability, and the ability to assess critical research questions in underrepresented populations. Further, patients engaged in prior research studies appeared to improve response rates, highlighting the importance of the researcher-participant relationship. Our work provides support for use of prenotifications via mail as well as incentives as critical methods to improve electronic survey response rates, particularly in traditionally hard-to-reach populations.

## Appendix

Multimedia Appendix 1 Demographic characteristics of pilot groups for respondents and all invited.

Multimedia Appendix 2 Response rates by specific pilot conditions, including a long versus condensed email, comparing prenotification approaches (letter versus postcard) and incentive types (gift to all individuals invited versus US $10 gift card for survey completion).

## Abbreviations

ADI: area deprivation index
