# Supplementary material for: Improving Electronic Survey Response Rates Among Cancer Center Patients During the COVID-19 Pandemic: Mixed Methods Pilot Study
Source: JMIR Cancer. 2021 Aug 6;7(3):e30265. doi: 10.2196/30265 (PMC8360334; doi:10.2196/30265)
Supplement: Multimedia Appendix 1 [file cancer_v7i3e30265_app1.docx]

| **Multimedia Appendix 1. Demographic characteristics of pilot groups for respondents and all invited** | | | | | | | | | | | | | | | | |
| --- | --- | --- | --- | --- | --- | --- | --- | --- | --- | --- | --- | --- | --- | --- | --- | --- |
|  | Long Email Only | | Email Only | | Email + Letter | | Email + Letter + Gift | | Email + Postcard | | Email + Gift card | | Email + Letter+ Gift card | | Email + Postcard+ Gift card | |
|  | Responded | Invited | Responded | Invited | Responded | Invited | Responded | Invited | Responded | Invited | Responded | Invited | Responded | Invited | Responded | Invited |
| n | 26 | 1,000 | 20 | 250 | 28 | 250 | 39 | 250 | 21 | 250 | 36 | 250 | 46 | 250 | 46 | 250 |
| Age, mean (sd) | 64.8 (11.4) | 64.3 (11.5) | 69.9 (9.7) | 64.3 (11.5) | 64.8 (11.3) | 64.0 (11.7) | 68.4 (10.2) | 65.4 (12.5) | 67.5 (10.6) | 65.6 (11.2) | 65.5 (10.7) | 64.3 (11.6) | 63.0 (12.5) | 63.8 (12.4) | 64.0 (9.8) | 64.9 (11.2) |
| Years Since Last Moffitt Visit, mean (sd) | 0.8 (1.1) | 1.6 (1.5) | 1.5 (1.7) | 1.6 (1.7) | 0.7 (0.8) | 1.6 (1.6) | 1.2 (1.3) | 1.6 (1.7) | 1.1 (1.5) | 1.5 (1.6) | 1.0 (1.5) | 1.5 (1.7) | 1.0 (1.4) | 1.4 (1.6) | 0.9 (1.3) | 1.3 (1.6) |
| Area Deprivation Index State Decile Rank, mean (sd) | 4.8 (2.4) | 5.2 (2.8) | 4.0 (2.6) | 5.0 (2.7) | 4.0 (2.3) | 4.7 (2.6) | 5.0 (2.9) | 5.1 (2.9) | 3.1 (2.2) | 4.9 (2.8) | 5.0 (2.7) | 5.2 (2.7) | 5.0 (2.6) | 4.9 (2.7) | 4.2 (2.4) | 5.0 (2.7) |
| Gender, n |  |  |  |  |  |  |  |  |  |  |  |  |  |  |  |  |
| Male | 8 | 461 | 10 | 114 | 19 | 125 | 16 | 136 | 7 | 120 | 16 | 131 | 21 | 136 | 21 | 116 |
| Female | 18 | 539 | 10 | 136 | 9 | 125 | 23 | 114 | 14 | 130 | 20 | 119 | 25 | 114 | 25 | 134 |
| Race, n |  |  |  |  |  |  |  |  |  |  |  |  |  |  |  |  |
| American Indian | 0 | 1 | 0 | 0 | 0 | 0 | 0 | 1 | 0 | 0 | 1 | 0 | 0 | 2 | 0 | 0 |
| Asian/Pacific Islander | 0 | 17 | 0 | 3 | 0 | 2 | 0 | 4 | 0 | 4 | 0 | 3 | 0 | 4 | 1 | 4 |
| Black | 0 | 125 | 1 | 28 | 4 | 38 | 2 | 37 | 2 | 35 | 3 | 39 | 7 | 37 | 7 | 40 |
| Other | 0 | 23 | 0 | 8 | 0 | 4 | 0 | 4 | 1 | 8 | 1 | 7 | 1 | 7 | 1 | 8 |
| White | 26 | 814 | 19 | 205 | 24 | 202 | 37 | 200 | 18 | 195 | 32 | 193 | 38 | 192 | 37 | 191 |
| Unknown | 0 | 20 | 0 | 6 | 0 | 4 | 0 | 4 | 0 | 8 | 0 | 8 | 0 | 8 | 0 | 7 |
| Ethnicity, n |  |  |  |  |  |  |  |  |  |  |  |  |  |  |  |  |
| Hispanic | 2 | 133 | 2 | 27 | 3 | 29 | 3 | 36 | 4 | 34 | 3 | 38 | 10 | 37 | 11 | 44 |
| Non-Hispanic | 24 | 846 | 18 | 220 | 25 | 217 | 36 | 208 | 17 | 209 | 33 | 205 | 36 | 206 | 35 | 201 |
| Unknown | 0 | 21 | 0 | 3 | 0 | 4 | 0 | 6 | 0 | 7 | 0 | 7 | 0 | 7 | 0 | 5 |
| Cancer Status, n |  |  |  |  |  |  |  |  |  |  |  |  |  |  |  |  |
| Invasive | 23 | 755 | 14 | 184 | 24 | 171 | 31 | 184 | 18 | 184 | 27 | 188 | 35 | 197 | 40 | 195 |
| Benign or In Situ | 1 | 70 | 1 | 23 | 1 | 23 | 2 | 12 | 1 | 15 | 1 | 18 | 2 | 15 | 0 | 17 |
| No cancer | 2 | 175 | 5 | 43 | 3 | 56 | 6 | 54 | 2 | 51 | 8 | 44 | 9 | 38 | 6 | 38 |
| Cancer Type at First Diagnosis, n ^a^ |  |  |  |  |  |  |  |  |  |  |  |  |  |  |  |  |
| Brain & Other Nervous System | 0 | 5 | 0 | 3 | 0 | 1 | 0 | 0 | 0 | 1 | 0 | 1 | 0 | 1 | 1 | 4 |
| Breast | 4 | 127 | 2 | 26 | 1 | 17 | 5 | 25 | 6 | 26 | 4 | 20 | 4 | 19 | 5 | 27 |
| Digestive System | 2 | 71 | 0 | 25 | 5 | 20 | 1 | 14 | 1 | 12 | 1 | 17 | 3 | 21 | 5 | 23 |
| Endocrine System | 0 | 13 | 0 | 1 | 0 | 3 | 0 | 3 | 1 | 5 | 0 | 4 | 1 | 9 | 1 | 4 |
| Gynecologic | 1 | 30 | 0 | 11 | 0 | 4 | 1 | 3 | 0 | 11 | 0 | 5 | 1 | 5 | 2 | 4 |
| Head & Neck | 0 | 31 | 1 | 12 | 1 | 7 | 0 | 3 | 0 | 7 | 2 | 7 | 1 | 7 | 0 | 8 |
| Hematologic | 4 | 99 | 6 | 23 | 2 | 26 | 5 | 27 | 5 | 32 | 7 | 29 | 5 | 27 | 9 | 35 |
| Male Genital | 2 | 105 | 1 | 16 | 6 | 22 | 7 | 27 | 2 | 18 | 5 | 28 | 3 | 26 | 1 | 24 |
| Melanoma | 1 | 37 | 1 | 11 | 4 | 15 | 2 | 15 | 0 | 8 | 1 | 10 | 4 | 14 | 1 | 12 |
| Neuroendocrine Tumor | 1 | 15 | 0 | 4 | 0 | 4 | 1 | 4 | 0 | 1 | 0 | 5 | 1 | 3 | 3 | 4 |
| Non-Melanoma Skin | 1 | 14 | 0 | 1 | 0 | 7 | 1 | 4 | 0 | 6 | 1 | 5 | 0 | 4 | 1 | 5 |
| Respiratory System | 2 | 63 | 2 | 12 | 1 | 12 | 3 | 13 | 1 | 12 | 2 | 19 | 0 | 12 | 0 | 11 |
| Urinary System | 1 | 45 | 1 | 11 | 2 | 11 | 2 | 17 | 0 | 15 | 0 | 12 | 6 | 16 | 5 | 9 |
| No Cancer at First Diagnosis | 2 | 58 | 0 | 17 | 2 | 12 | 1 | 18 | 2 | 14 | 1 | 8 | 4 | 16 | 4 | 16 |
| Metastatic at First Diagnosis | 1 | 16 | 0 | 5 | 0 | 3 | 1 | 5 | 0 | 5 | 1 | 4 | 1 | 8 | 0 | 1 |
| Uncertain Type | 1 | 26 | 0 | 6 | 0 | 7 | 1 | 6 | 0 | 11 | 2 | 14 | 1 | 9 | 2 | 8 |
| Stage at First Diagnosis, n ^a^ |  |  |  |  |  |  |  |  |  |  |  |  |  |  |  |  |
| 0 | 1 | 33 | 1 | 7 | 1 | 2 | 0 | 7 | 1 | 7 | 1 | 6 | 1 | 5 | 3 | 10 |
| 1 | 6 | 180 | 4 | 34 | 8 | 51 | 8 | 42 | 5 | 39 | 6 | 39 | 12 | 47 | 15 | 44 |
| 2 | 3 | 102 | 3 | 27 | 8 | 29 | 3 | 22 | 2 | 26 | 2 | 25 | 4 | 20 | 3 | 29 |
| 3 | 1 | 67 | 2 | 24 | 2 | 12 | 4 | 18 | 1 | 16 | 0 | 9 | 3 | 16 | 3 | 21 |
| 4 | 1 | 56 | 0 | 13 | 2 | 11 | 7 | 17 | 2 | 11 | 2 | 13 | 2 | 20 | 1 | 16 |
| Unknown | 11 | 317 | 4 | 79 | 3 | 66 | 9 | 78 | 7 | 85 | 16 | 96 | 13 | 89 | 15 | 75 |
| ^a^ among those with a cancer diagnosis | | | | |  |  |  |  |  |  |  |  |  |  |  |  |
