# Supplementary material for: Improving Electronic Survey Response Rates Among Cancer Center Patients During the COVID-19 Pandemic: Mixed Methods Pilot Study
Source: JMIR Cancer. 2021 Aug 6;7(3):e30265. doi: 10.2196/30265 (PMC8360334; doi:10.2196/30265)
Supplement: Multimedia Appendix 2 [file cancer_v7i3e30265_app2.docx]

| **Multimedia Appendix 2. Response rates by specific pilot conditions, including a long versus condensed email, comparing pre-notification approaches (letter versus postcard) and incentive types (gift to all individuals invited vs. $10 gift card for survey completion)** | | | | | |
| --- | --- | --- | --- | --- | --- |
|  | Long email (n=26/1000) | | Short email (n=20/250) | | OR (95% CI)^a^ |
|  | n (%) complete | n (%) incomplete | n (%) complete | n (%) incomplete |  |
| Overall | 26 (2.6) | 974 (97.4) | 20 (8.0) | 230 (92.0) | **3.26 (1.79, 5.94)** |
| White | 26 (3.1) | 808 (96.9) | 19 (9.0) | 192 (91.0) | **3.08 (1.67, 5.67)** |
| Non-White | 0 (0.0) | 166 (100.0) | 1 (2.6) | 38 (97.4) | n/a |
| Non-Hispanic | 24 (2.8) | 843 (97.2) | 18 (8.1) | 205 (91.9) | **3.08 (1.64, 5.79)** |
| Hispanic | 2 (1.5) | 131 (98.5) | 2 (7.4) | 25 (92.6) | 5.24 (0.70, 38.96) |
| Male | 8 (1.7) | 453 (98.3) | 10 (8.8) | 104 (91.2) | **5.44 (2.10, 14.13)** |
| Female | 18 (3.3) | 521 (96.7) | 10 (7.4) | 126 (92.7) | **2.30 (1.04, 5.10)** |
| <2 years since last visit | 23 (3.3) | 670 (96.7) | 14 (8.1) | 158 (91.9) | **2.58 (1.30, 5.13)** |
| 2-5 years since last visit | 3 (1.0) | 304 (99.0) | 6 (7.7) | 72 (92.3) | **8.44 (2.06, 34.57)** |
| < 65 years old | 13 (2.8) | 458 (97.2) | 5 (4.1) | 117 (95.9) | 1.51 (0.53, 4.31) |
| ≥ 65 years old | 13 (2.5) | 516 (97.5) | 15 (11.7) | 113 (88.3) | **5.27 (2.44, 11.38)** |
| Any Cancer | 23 (3.1) | 732 (97.0) | 14 (7.6) | 170 (92.4) | **2.62 (1.32, 5.20)** |
| Benign, In Situ, or No Cancer | 3 (1.2) | 242 (98.8) | 6 (9.1) | 60 (90.9) | **8.07 (1.97, 33.19)** |
| ADI Rank 1-5 | 16 (2.9) | 537 (97.1) | 14 (9.9) | 127 (90.1) | **3.70 (1.76, 7.78)** |
| ADI Rank 6-10 | 8 (1.9) | 409 (98.1) | 5 (5.1) | 94 (95.0) | 2.72 (0.87, 8.50) |
|  |  |  |  |  |  |
|  | Email + Letter (n=28/250) | | Email + Postcard (n=21/250) | | OR (95% CI)^b^ |
|  | n (%) complete | n (%) incomplete | n (%) complete | n (%) incomplete |  |
| Overall | 28 (11.2) | 222 (88.8) | 21 (8.4) | 229 (91.6) | 0.73 (0.40, 1.32) |
| White | 24 (11.7) | 182 (88.4) | 18 (8.9) | 185 (91.1) | 0.74 (0.39, 1.41) |
| Non-White | 4 (9.1) | 40 (90.9) | 3 (6.4) | 44 (93.6) | 0.68 (0.14, 3.23) |
| Non-Hispanic | 25 (11.3) | 196 (88.7) | 17 (7.9) | 199 (92.1) | 0.67 (0.35, 1.28) |
| Hispanic | 3 (10.3) | 26 (89.7) | 4 (11.8) | 30 (88.2) | 1.16 (0.24, 5.65) |
| Male | 19 (15.2) | 106 (84.8) | 7 (5.8) | 113 (94.2) | **0.35 (0.14, 0.86)** |
| Female | 9 (7.2) | 116 (92.8) | 14 (10.8) | 116 (89.2) | 1.56 (0.65, 3.74) |
| <2 years since last visit | 25 (15.3) | 138 (84.7) | 17 (9.9) | 155 (90.1) | 0.61 (0.31, 1.17) |
| 2-5 years since last visit | 3 (3.5) | 84 (96.6) | 4 (5.1) | 74 (94.9) | 1.51 (0.33, 6.98) |
| < 65 years old | 13 (10.8) | 107 (89.2) | 6 (5.6) | 102 (94.4) | 0.48 (0.18, 1.32) |
| ≥ 65 years old | 15 (11.5) | 115 (88.5) | 15 (10.6) | 127 (89.4) | 0.91 (0.42, 1.93) |
| Any Cancer | 24 (14.0) | 147 (86.0) | 18 (9.8) | 166 (90.2) | 0.66 (0.35, 1.27) |
| Benign, In Situ, or No Cancer | 4 (5.1) | 75 (94.9) | 3 (4.6) | 63 (95.5) | 0.89 (0.19, 4.14) |
| ADI Rank 1-5 | 21 (13.8) | 131 (86.2) | 17 (12.0) | 125 (88.0) | 0.84 (0.43, 1.68) |
| ADI Rank 6-10 | 6 (6.5) | 87 (93.6) | 3 (3.1) | 94 (96.9) | 0.46 (0.11, 1.91) |
|  |  |  |  |  |  |
|  | Gift incentive (n=39/250) | | Gift card incentive (n=128/750) | | OR (95% CI)^c^ |
|  | n (%) complete | n (%) incomplete | n (%) complete | n (%) incomplete |  |
| Overall | 39 (15.6) | 211 (84.4) | 128 (17.1) | 622 (82.9) | 1.11 (0.75, 1.65) |
| White | 37 (18.1) | 167 (81.9) | 107 (17.9) | 492 (82.1) | 0.98 (0.65, 1.48) |
| Non-White | 2 (4.4) | 44 (95.7) | 21 (13.9) | 130 (86.1) | 3.55 (0.80, 15.77) |
| Non-Hispanic | 36 (16.8) | 178 (83.2) | 104 (16.5) | 527 (83.5) | 0.98 (0.64, 1.48) |
| Hispanic | 3 (8.3) | 33 (91.7) | 24 (20.2) | 95 (79.8) | 2.78 (0.79, 9.84) |
| Male | 16 (11.8) | 120 (88.2) | 58 (15.1) | 325 (84.9) | 1.34 (0.74, 2.42) |
| Female | 23 (20.2) | 91 (79.8) | 70 (19.1) | 297 (80.9) | 0.93 (0.55, 1.58) |
| <2 years since last visit | 29 (17.3) | 139 (82.7) | 104 (19.7) | 424 (80.3) | 1.18 (0.75, 1.85) |
| 2-5 years since last visit | 10 (12.2) | 72 (87.8) | 24 (10.8) | 198 (89.2) | 0.87 (0.40, 1.91) |
| < 65 years old | 12 (11.2) | 95 (88.8) | 66 (18.2) | 297 (81.8) | 1.76 (0.91, 3.39) |
| ≥ 65 years old | 27 (18.9) | 116 (81.1) | 62 (16.0) | 325 (84.0) | 0.82 (0.50, 1.35) |
| Any Cancer | 31 (16.9) | 153 (83.2) | 102 (17.6) | 478 (82.4) | 1.05 (0.68, 1.64) |
| Benign, In Situ, or No Cancer | 8 (12.1) | 58 (87.9) | 26 (15.3) | 144 (84.7) | 1.31 (0.56, 3.06) |
| ADI Rank 1-5 | 25 (17.2) | 120 (82.8) | 79 (18.4) | 350 (81.6) | 1.08 (0.66, 1.78) |
| ADI Rank 6-10 | 14 (14.9) | 80 (85.1) | 43 (14.9) | 245 (85.1) | 1.00 (0.52, 1.93) |
| Abbreviations: ADI=Area Deprivation Index, OR=Odds Ratio, CI=Confidence Interval | | | | | |
| ^a^ Odds of completing the survey if a short email was sent versus a long email | | | | | |
| ^b^ Odds of completing the survey if a postcard was included versus a letter | | | | | |
| ^c^ Odds of completing the survey if the incentive was a gift card versus a gift | | | | | |
